# 1st Global Consensus for Clinical Guidelines: Identifying a Core Outcome Set for Implant Dentistry in Edentulous Maxilla Rehabilitation

**DOI:** 10.1111/clr.70075

**Published:** 2026-02-24

**Authors:** Giulia Brunello, Guo‐Hao Lin, Ina Kopp, Alonso Carrasco‐Labra, Ronald E. Jung, Hom‐Lay Wang, Frank Schwarz, Franz J. Strauss

**Affiliations:** ^1^ Department of Oral Surgery University Hospital of Düsseldorf Düsseldorf Germany; ^2^ Department of Orthodontics and Dentofacial Orthopaedics Charité—Universitätsmedizin Berlin, Freie Universität Berlin and Humboldt‐Universität zu Berlin Berlin Germany; ^3^ Department of Orofacial Sciences University of California San Francisco School of Dentistry San Francisco California USA; ^4^ Department of Periodontics & Oral Medicine University of Michigan School of Dentistry Ann Arbor Michigan USA; ^5^ AWMF‐Institut für Medizinisches Wissensmanagement Philipps‐Universität Marburg Marburg Germany; ^6^ Department of Preventive and Restorative Sciences, School of Dental Medicine, Center for Integrative Global Oral Health University of Pennsylvania Philadelphia Pennsylvania USA; ^7^ Clinic of Reconstructive Dentistry, Center of Dental Medicine University of Zurich Zurich Switzerland; ^8^ Department of Oral Surgery, Implantology and Oral Medicine Goethe University Frankfurt am Main Germany; ^9^ Universidad Autonoma de Chile Santiago Chile

**Keywords:** consensus conference, core outcome set, Delphi methodology, dental implants

## Abstract

**Objectives:**

To identify a core outcome set (COS) to standardize outcome measures for studies on the rehabilitation of the edentulous maxilla in implant dentistry. This work was conducted in preparation for the 1st Global Consensus for Clinical Guidelines (GCCG) for the Rehabilitation of the Edentulous Maxilla.

**Materials and Methods:**

The COS was developed using a standardized and validated methodology, following the Core Outcome Measures in Effectiveness Trials (COMET) initiative. Outcomes were first identified through eight systematic reviews covering surgical and prosthetic aspects of edentulous maxilla rehabilitation. This preliminary list was expanded by input from clinical and cross‐disciplinary experts as well as patient surveys to capture additional relevant outcomes that were not represented in the literature. A 3‐round Delphi survey was conducted. The first round was conducted prior to the meeting and the second and third round took place during the in‐person consensus meeting. In the first round, participants could propose new outcomes and rated each item using a 9‐point Likert scale, from 1 (not important) to 9 (critical), with scores of 7–9 denoting critical importance, 4–6 importance, and 1–3 low importance for decision making. In the second round, participants re‐scored a revised outcome list incorporating written suggestions and consolidated overlapping items from the first round. Outcomes rated 7–9 by at least 75% of participants in the second round constituted the final COS selection. In the third round, these selected outcomes were evaluated for their relevance across the different stages of the GCCG workflow: patient selection, diagnostic tools, treatment planning, treatment procedure, management of complications during the treatment procedure, maintenance, and management of complications during maintenance.

**Results:**

Of the 105 experts invited, response rates for the three Delphi rounds were 87.6%, 95.2%, and 92.4%, respectively. An initial list of 119 outcomes was generated from systematic reviews and surveys and subsequently refined after the first round based on participant feedback. In the second round, 34 outcomes were rated as critical and included in the final COS. These comprised 10 patient‐reported outcomes (PROs): aesthetic satisfaction; chewing function/comfort/discomfort; complications during treatment/maintenance; ease of cleaning/oral hygiene efficacy; pain; patient overall satisfaction with treatment; patient‐reported complaints; prosthesis retention/stability; quality of life; speech/phonetics/pronunciation function, 22 objective clinician‐reported outcomes (ClinROs): biological complications; history of patient compliance; implant failure; implant primary stability; implant success; implant survival; mechanical/technical complications; peri‐implant health (implant level); peri‐implant health (patient level); peri‐implant mucositis; peri‐implant suppuration; peri‐implantitis; plaque index/oral hygiene; postoperative complications; presence of keratinized mucosa; prosthesis failure; prosthesis success; prosthetic complications; radiographic marginal bone level; radiographic marginal bone loss; surgical/intraoperative complications; width of keratinized mucosa and two subjective ClinROs (clinician's treatment success; prosthodontic maintenance events/complications). Additional outcomes proposed during the second round were excluded since they represented general comments, process indicators, or alternative wording of outcomes already considered. In the third round, 21 COS outcomes were deemed relevant to the maintenance and/or post‐treatment complication management phases, 3 were deemed relevant before treatment, and another 3 were deemed relevant only during treatment.

**Conclusion:**

The proposed COS for studies on the rehabilitation of the edentulous maxilla in implant dentistry was developed using the COMET methodology. Although patients' perspectives were incorporated during the preliminary survey phase, they did not participate directly in the Delphi, highlighting an opportunity for future research to further enhance patient involvement in COS development. The widespread implementation of this COS is expected to improve the consistency of outcome reporting and facilitate comparisons of the effectiveness of current and emerging interventions.

## 1 Introduction

Selecting appropriate outcomes in clinical trials and systematic reviews is crucial to ensure that research findings are meaningful, reliable and relevant to patients, clinicians and other key stakeholders. An outcome is defined as a change in patient status over time, whereas an outcome measure is a tool or test used to assess a patient's status over time (Fetters and Tilson 2018). In clinical trials, an outcome is a variable measured to evaluate the effects of an intervention on participants (Butcher et al. 2022b, 2022a), thereby determining its efficacy and safety. Inconsistencies in reported outcomes hinder the ability to compare study results and limit the feasibility of meta‐analysis, which is vital for strengthening statistical power and guiding evidence‐based practice. To address this issue, core outcome sets (COS) have been introduced (Williamson et al. 2012).

A COS is a consensus‐based set of outcomes that should be measured and reported, at a minimum, in all clinical trials within a specific healthcare area (Williamson et al. 2012). Primary aims of COS are to enhance comparability across trials, reduce selective outcome reporting and ensure that outcomes reflect the priorities of both patients and professionals. Moreover, COS play a critical role in the development of clinical practice guidelines, supporting recommendations based on relevant evidence (Gorst et al. 2016).

In implant dentistry, efforts to standardize minimum set of outcomes are underway (Tonetti, Heitz‐Mayfield, et al. 2023). However, implant dentistry is a multidisciplinary field involving prosthodontists, periodontists, oral and maxillofacial surgeons and general practitioners. It also encompasses a wide variety of clinical scenarios such as the rehabilitation of partially or fully edentulous arches in the maxilla, mandible, or both. As such, earlier COS efforts, though essential, require further refinement to address this diversity in practice and perspective. Thus, it is anticipated that COS will need to evolve over time to reflect specific conditions and treatment contexts.

The edentulous maxilla is a debilitating condition with significant negative impacts on both oral and general health (Al‐Rafee 2020; Fagundes et al. 2024; Gennai et al. 2022). Treatment options using dental implants for full‐arch restorations vary widely in terms of number, length, and location. Although the conventional treatment for an edentulous maxilla is a complete denture, various implant‐based approaches using standard‐length, short, or zygomatic implants are increasingly being used (Al‐Nawas et al. 2023; Jung et al. 2018). Rehabilitation of the edentulous maxilla is often more invasive and clinically complex (Khoury and Hanser 2019; Urban et al. 2019) compared to treatments for single‐tooth replacement in partially edentulous dentition (Yao et al. 2018).

The expanding body of clinical research in implant dentistry has resulted in the use of a wide variety of outcome measures, many of which lack standardization. This inconsistency limits the comparability of studies and hinders the ability to synthesize findings in systematic reviews. Therefore, the development of a dedicated COS for the edentulous maxilla is essential. A well‐defined COS would promote more consistent and meaningful outcome reporting, ultimately benefiting clinicians, researchers, and patients alike.

This work was conducted in preparation for the 1st Global Consensus for Clinical Guidelines (GCCG) for the Rehabilitation of the Edentulous Maxilla. A detailed description of the GCCG consensus process is provided in the accompanying summary manuscript (Schwarz et al. 2026).

## 2 Materials and Methods

The present study was registered with the COMET Initiative (No. 3559 accessible at https://www.comet‐initiative.org/Studies/Details/3559) and reported following the Core Outcome Set–STAndards for Reporting (COS‐STAR) Statement (Kirkham et al. 2017).

### 2.1 Core Outcome Set Development Group

The Scientific Chairs (F.S., H.‐L.W.) and methodologist (I.K.) of the GCCG, with the support of clinical experts (G.B., G.‐H.L., R.E.J., F.J.S., A.C.L.), led the development of the COS, including the coordination of the Delphi consensus process.

### 2.2 Project Development

The project comprised two main phases.

In the first phase, systematic reviews were conducted to identify candidate outcomes relevant to the rehabilitation of the edentulous maxilla. Eight systematic reviews were conducted to identify, categorize, and critically appraise the use and methodological quality of patient‐reported outcomes (PROs) and clinician‐reported outcomes (ClinROs) in studies addressing surgical and prosthetic interventions. These reviews also summarized contemporary practices and reporting trends over the past decade.

To complement these findings, six structured surveys were administered with clinical experts, cross‐disciplinary experts, and patients. The clinical expert surveys collected input from 472 clinicians across multiple countries, focusing mainly on ClinRO‐related decision‐making domains. In contrast, the patient and cross‐disciplinary surveys explored patient‐centered outcomes such as expectations, treatment preferences, perceived burdens, and priorities related to esthetics, comfort, function, and psychosocial well‐being.

In the second phase, an international Delphi survey was utilized to achieve consensus among participants and finalize the core outcomes to be included in future clinical trials.

### 2.3 Scope of the Core Outcome Set

*Health condition*: complete edentulism of the maxilla requiring implant‐supported rehabilitation. *Population*: adults eligible for full‐arch implant therapy in the maxilla.

*Interventions/comparators*: surgical and prosthetic strategies encompassed by the GCCG working groups (e.g., standard‐length, short, and zygomatic implants; fixed vs. removable prostheses; immediate/early/conventional loading; timing of placement; augmentation procedures) (Donos et al. 2026; Fiorellini et al. 2026; Pala et al. 2026; Stilwell et al. 2026).

*Settings*: specialty and general practice centers providing implant rehabilitation.

This scope defined eligibility criteria for outcomes considered during long‐listing and Delphi prioritization stages.

### 2.4 Phase 1—Identification of Candidate Outcomes

Systematic reviews were conducted with the purpose of identifying patient‐reported outcomes (PROs) and clinician‐reported outcomes (ClinROs) and their respective assessment tools, i.e., patient‐reported outcome measures (PROMs) and clinician‐reported outcome measures (CROMs). Two reviewers (G.B. and F.J.S.) independently extracted outcome labels from each review; discrepancies were resolved by consensus. Survey‐generated items were mapped against review‐derived items; duplicates were merged under a standardized label following COMET guidance, with representative synonyms retained with the purpose of the text to preserve nuance.

In addition, six structured surveys, targeting clinical experts (Brunello et al. 2026; Lin, Strauss, et al. 2026; Schoenbaum et al. 2026; Strauss et al. 2026), cross‐disciplinary experts and patients (Lin, Brunello, et al. 2026) were conducted to identify outcomes relevant to both patients and clinicians not addressed in the current literature. All these outcomes were considered for prioritization during the Delphi consensus process (Schwarz et al. 2026).

The combined dataset from systematic reviews and surveys was refined into a preliminary shortlist by consolidating similar or overlapping outcomes (Tables S1–S3). The rationalization, conducted by the COS Development Group, ensured that each summary outcome retained the intent and detail of the original, more granular data. Care was taken to avoid excessive condensation at this stage, thereby preserving the diversity of the original input. These outcomes were then organized into three groups: PROs, objective ClinROs and subjective ClinROs.

*Patient‐reported outcome (PRO)*: any report of the status of a patient's health condition that comes directly from the patient without interpretation of the patient's response by a clinician or anyone else (U.S. Food and Drug Administration, F 2021).

*Clinician‐reported outcome (ClinRO)*: a type of clinical outcome assessment based on a report that comes from a trained health‐care professional after observation of a patient's health condition. Most CROMs involve a clinical judgment or interpretation of the measurable changes in a patient or participant's symptoms, overall health, ability to function, quality of life or survival (FDA‐NIH Biomarker Working Group 2016). A CROM can be defined as a dichotomous reading, a scalar or categorical rating (Powers 3rd et al. 2017).

Within this framework, objective outcomes were characterized as those measurable with high reproducibility and minimal interpretive variability, including parameters such as implant survival, presence of keratinized mucosa, and marginal bone level changes. In contrast, the classification of “subjective” outcomes was pragmatically introduced within the scope of the GCCG initiative to denote outcomes based on the clinician's perception of treatment success. It is recognized that this terminology is not formally defined within the COMET Handbook or existing COS literature (Williamson et al. 2017).

### 2.5 Phase 2—Delphi Consensus

A three‐round Delphi process was conducted, with the first round conducted prior to the meeting and the second and third round taking place during the in‐person consensus meeting (over a 3‐day period) via an online survey link. The Delphi method consists of a series of anonymous questionnaires completed by a panel of carefully selected experts (Khodyakov et al. 2023). In the first round, responses are based on each expert's knowledge and personal experience. In subsequent rounds, participants are shown how their answers compare with those of other panelists, enabling them to reconsider their ratings in light of the group's feedback (Khodyakov et al. 2023). Since there is no direct communication between participants, this structured feedback serves as a mechanism to reconcile differing stakeholder opinions and is therefore critical to achieving consensus (Williamson et al. 2017).

The Delphi technique is a widely applied method for reaching consensus on core outcome domains (Bova et al. 2023; Kottner et al. 2018; Tonetti, Sanz, et al. 2023; Williamson et al. 2017). Its key advantages are that participants can independently and anonymously assess the importance of outcomes, and that it enables the inclusion of large, geographically diverse groups such as the GCCG. For the rating process, nine‐point scales were used, as originally proposed by the RAND Appropriateness Method (Khodyakov et al. 2023), together with the GRADE approach (Guyatt et al. 2011), which is recommended in COS development to guide consensus and outcome selection (De Meyer et al. 2019).

Preliminary information about the COS and the consensus procedure was shared during online meetings with the full group of delegates, as well as in separate sessions for each of the four working groups in the weeks preceding the 1st GCCG meeting in Boston, June 16–18, 2025.

**FIGURE 1:**
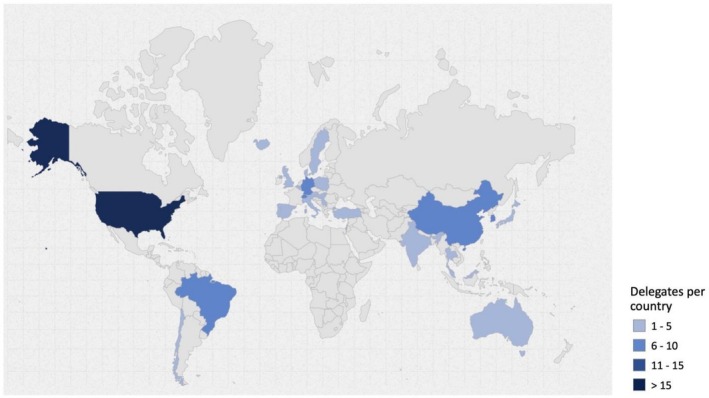
Distribution by country of the 105 delegates. Map created using the open‐source online tool available at https://www.amcharts.com/ (amCharts, Neringa, Lithuania).

The 105 delegates attending the Boston meeting were invited to participate in the anonymous Delphi surveys aimed at assessing and prioritizing outcomes to define the COS for edentulous maxilla rehabilitation. Geographical distribution of the delegates is presented in Figure 1. Details of the delegates are provided in Schwarz et al. (2026).

The European Association for Osseointegration (EAO) Office distributed the survey links via email. Comprehensive information about the study was provided at each round in the invitation email and on the introductory page of the anonymous online Delphi survey. The surveys were conducted electronically using SurveyMonkey (San Mateo, CA, USA), with all data collected, stored, and processed anonymously. Completion of the anonymous questionnaires was regarded as part of the delegates' responsibilities, with survey submission considered as implied consent to participate.

#### 2.5.1 First‐Round Delphi Survey

The first‐round survey was distributed to participants via email on June 6, 2025. To reduce the risk of the survey email being filtered into spam folders, a separate notification email, without the survey link, was sent shortly after the initial distribution. A follow‐up reminder containing the survey link was sent on June 9, 2025. The survey closed on June 11, 2025.

Participants were asked to rate each outcome using the 9‐point Likert scale proposed by the GRADE working group (Guyatt et al. 2011; McCaul et al. 2025). On this scale, scores of 1 to 3 reflected outcomes of low importance, scores of 4 to 6 indicated outcomes that were important but not critical, and scores of 7 to 9 represented outcomes considered critical for decision‐making.

In the first round of the Delphi process, outcomes were organized into three predefined groups (1. PROs, 2. objective ClinROs and 3. subjective ClinROs) and presented alphabetically within each category to minimize potential question order bias (De Meyer et al. 2019; O'Malley et al. 2023; Zanjir et al. 2025). This strategy aimed to reduce the possibility that the sequence of outcomes might influence how participants assigned importance ratings. A total of 119 outcomes were assessed, including 40 PROs, 69 objective ClinROs, and 10 subjective ClinROs (Appendix S1). Participants were also invited to propose additional outcomes they considered relevant to the rehabilitation of the edentulous maxilla but that were not included in the initial list. Outcomes were considered critically important in the first round if at least 75% of respondents rated them as critical (scores 7–9).

#### 2.5.2 Second‐Round Delphi Survey

The second‐round survey was distributed to participants via email on the evening (EST) of Sunday, June 15, 2025. Along with the survey link, participants received a summary of the results of the first round (% scores 7–9) and a detailed explanation of the revision process. To further support transparency and engagement, members of the COS Development Group delivered a presentation outlining the COS development process and the first‐round findings during the plenary morning session on Monday, June 16, 2025. The survey remained open until the afternoon of the same day. Although the survey link was distributed during the in‐person event, all ratings were submitted individually through an anonymous online platform; no live or public voting occurred.

In the second round, participants were asked to re‐score a revised version of the original first‐round questionnaire using the same 9‐point Likert scale. Revisions were made based on the comments and suggestions received during the first round. These included clarifying outcome definitions, removing non‐outcome items (such as process indicators), and incorporating additional outcomes proposed by respondents. Outcomes identified as repetitive, irrelevant, or not representing valid PROs or ClinROs were excluded. In addition, overlapping outcomes were consolidated under a single standardized term, following the COMET guidelines (Williamson et al. 2017). Where necessary, descriptions were added to enhance the clarity of specific outcomes.

This iterative process ensured that the outcome selection for the final core set was guided by both empirical data and expert consensus, leading to a refined and clinically meaningful set of outcomes for use in patient‐centered research on the rehabilitation of the edentulous maxilla.

In the second round, participants evaluated a total of 109 outcomes, including 41 PROs, accompanied where appropriate by related PROMs for clarification, 60 objective ClinROs, and 8 subjective ClinROs (Appendix S2). The Round‐2 list (*n* = 109) was generated by: (a) removing non‐outcome/process items; (b) consolidating overlapping outcomes under a standardized term; and (c) clarifying definitions for ambiguous items. Round‐2 represented a refined set rather than only Round‐1 outcomes meeting the ≥ 75% threshold. To facilitate evaluation, the objective ClinROs were further divided into categories, as previously described for the earlier COS in implant dentistry (Sanz et al. 2023): implant performance, implant‐supported prosthesis performance, surgical domain, and peri‐implant tissue health. Outcomes were presented in alphabetical order within each group of outcomes and objective ClinROs category to minimize potential bias caused by the order of questions. Participants were also given the possibility to suggest any additional outcomes they felt were missing from the revised questionnaire.

To assess the level of consensus regarding the importance of each outcome in the second round, critical for decision making were considered those outcomes rated 7–9 by ≥ 75% of respondents.

#### 2.5.3 Third‐Round Delphi Survey

Findings from the second round (percentages of scores 7–9) were summarized by the COS Development Group during the plenary afternoon session on Monday, June 16, 2025. The results were also provided to participants as a document, along with the third‐round survey, which was sent via email on the evening of the same day. The survey remained open until the afternoon of the following day. Although the survey link was distributed during the in‐person event, all ratings were submitted individually through an anonymous online platform; no live or public voting occurred.

The third round focused on outcomes identified as *critically important* in the second round (≥ 75% of participants rating 7–9) by at least 75% of participants. These outcomes were evaluated for their relevance within the different stages of the GCCG workflow. Participants evaluated 34 outcomes in total: 10 PROs, accompanied, where appropriate, by related PROMs for clarification, 22 objective ClinROs, and 2 subjective ClinROs (Appendix S3). Outcomes were presented alphabetically within each category to minimize bias from question sequence.

Participants were asked to indicate at which of the following stages each outcome was relevant (multiple selections were allowed):

- Patient selection
- Diagnostic tools
- Treatment planning
- Treatment procedure
- Complications during treatment
- Maintenance
- Complications during maintenance

The first three stages represented the pre‐treatment phase. While outcomes could be relevant to multiple pre‐treatment stages, this did not imply that they needed to be recorded multiple times during that phase. The subsequent two stages related to the treatment phase, and the final two stages pertained to post‐treatment.

Outcomes were considered relevant to a specific phase of the GCCG workflow if they were selected by at least 75% of participants for that stage.

### 2.6 Analysis

Each survey response was analyzed individually using descriptive statistics, with results reported as the percentage of participants assigning scores within each range of the 9‐point Likert scale. In addition, the median and interquartile range (IQR) were calculated for each outcome, following the RAND guidelines (Khodyakov et al. 2023).

For the multiple‐choice, multiple‐selection questions in the third round, the percentage of participants who selected each GCCG workflow stage was calculated and reported. To minimize the risk of data dispersion across the pre‐treatment stages, *Patient Selection*, *Diagnostic Tools*, and *Treatment Planning* were analyzed both individually and as a combined group. In the pooled analysis, an outcome was considered relevant to the pre‐treatment phase by a participant if it was selected in at least one of the three stages. Outcomes were selected primarily using the ≥ 75% rating of 7–9. Medians and IQRs were summarized to appraise dispersion and guide clarifications or refinements in Round 2; dispersion statistics were not used as exclusion criteria. Data analysis was conducted using STATA version 18, and GraphPad Prism version 10.

## 3 Results

Phase 1 (evidence mapping and surveys) identified 119 candidate outcomes (40 PROs, 69 objective ClinROs, 10 subjective ClinROs) for Round 1, based on eight systematic reviews (Francisco et al. 2026; Lin, Chen, et al. 2026; Pannuti et al. 2026; Park et al. 2026; Romito et al. 2026; Sabri et al. 2026; Saleh et al. 2026; Thoma et al. 2026) and six targeted surveys (Brunello et al. 2026; Lin, Chen, et al. 2026; Lin, Strauss, et al. 2026; Schoenbaum et al. 2026; Strauss et al. 2026). These surveys captured outcomes reported by clinicians, patients, and cross‐disciplinary experts.

### 3.1 First‐Round Delphi Survey

Of 105 delegates, 92 completed the survey, yielding a response rate of 87.6%. Prioritization of outcomes is presented in Tables S1–S3. Using the ≥ 75% rating 7–9 criterion, 13 PROs, 27 objective ClinROs and 5 subjective ClinROs were considered of critical importance. Median and IQR obtained for each outcome are reported in Figures S1–S6. Thirty participants suggested additional outcomes; four suggested only PROs, four proposed only ClinROs, and 22 both.

### 3.2 Second‐Round Delphi Survey

Of 105 delegates, 100 participated in the survey, resulting in a response rate of 95.2%. The outcome prioritization is presented in Tables S4–S6, with corresponding medians and IQRs shown in Figures S4–S6. Outcomes rated *critically important* (≥ 75% of respondents scoring 7–9) were selected for inclusion in the COS. The resulting COS for reporting on rehabilitation of the edentulous maxilla comprises the following 10 PROs, 22 objective ClinROs and 2 subjective ClinROs:

*PROs*

- Aesthetic satisfaction
- Chewing function/comfort/discomfort
- Complications during treatment/maintenance
- Ease of cleaning/oral hygiene efficacy
- Pain
- Patient overall satisfaction with treatment
- Patient‐reported complaints
- Prosthesis retention/stability
- Quality of life (Oral Health‐Related Quality of Life, OHRQoL)
- Speech/phonetics/pronunciation function

*Objective ClinROs*

- Implant performance
  ○ Implant failure
  ○ Implant success
  ○ Implant survival
  ○ Mechanical/technical complications
- Implant‐supported prosthesis performance
  ○ Plaque index/Oral hygiene
  ○ Prosthesis failure
  ○ Prosthesis success
  ○ Prosthetic complications
- Surgical domain
  ○ Implant primary stability
  ○ Postoperative complications
  ○ Presence of keratinized mucosa
  ○ Radiographic marginal bone level
  ○ Radiographic marginal bone loss
  ○ Surgical/intraoperative complications
  ○ Width of keratinized mucosa
- Peri‐implant tissue health
  ○ Biological complications
  ○ History of patient compliance
  ○ Peri‐implant health (implant level)
  ○ Peri‐implant health (patient level)
  ○ Peri‐implant mucositis
  ○ Peri‐implant suppuration
  ○ Peri‐implantitis

*Subjective ClinROs (clinicians' perception)*

- Clinician's treatment success
- Prosthodontic maintenance events/complications

Thirteen participants responded to the open‐ended questions with suggestions for additional outcomes: four proposed only PROs, three suggested only ClinROs, and six recommended both. Most of these suggestions were general comments, process indicators, or alternative wording of outcomes already evaluated.

### 3.3 Third‐Round Delphi Survey

In round 3, outcomes selected as *critical* in round 2 were mapped to GCCG workflow stages. Here, 97 out of 105 delegates responded to the survey, yielding a response rate of 92.4%. Details are provided in Tables S7–S9.

When the three pre‐operative stages of the GCCG workflow (Patient selection, Diagnostic tools, Treatment planning) were analyzed separately, no outcomes reached the 75% threshold, except the history of patient compliance (patient selection). However, when these three stages were pooled into a single composite category (“pre‐op”) and dichotomized into a binary outcome, the resulting agreement percentages increased. Outcomes exceeding the 75% threshold included: Aesthetic satisfaction (87.6%), plaque index/oral hygiene (78.3%), and history of patient compliance (81.4%).

Near‐threshold outcomes included chewing function/comfort/discomfort (74.2%), quality of life (71.1%), speech/phonetics/pronunciation function (74.2%) and presence of keratinized mucosa (73.2%).

During the *treatment phase*, pain (PRO) and implant primary stability (objective ClinRO) exceeded the 75% threshold and postoperative complications surpassed the cutoff in the *management of complications during* the *treatment procedure*.

During maintenance, several outcomes met the threshold:

– Two PROs: ease of cleaning/oral hygiene efficacy and patient overall satisfaction with treatment,
– Fourteen objective ClinROs: implant success, implant survival, mechanical/technical complications, plaque index/oral hygiene, prosthesis success, prosthetic complications, radiographic marginal bone level, radiographic marginal bone loss, biological complications, peri‐implant health (implant level), peri‐implant health (patient level), peri‐implant mucositis, peri‐implant suppuration; peri‐implantitis
– Two subjective ClinROs: clinician's treatment success and prosthodontic maintenance events/complications.

For the *management of complications during the maintenance* phase, the following reached the threshold:

– One PRO: complications treatment/maintenance
– Six objective ClinROs: implant failure, mechanical/technical complications, prosthesis failure; prosthetic complications, peri‐implant suppuration, peri‐implantitis
– One subjective ClinRO: prosthodontic maintenance events/complications

A simplified summary list of COS outcomes by workflow stages is presented in Table 1, where “Pre‐op” represents the pooled analysis, where an outcome was considered relevant to the pre‐treatment phase by a participant if it was selected in at least one of the pre‐treatment stages.

**TABLE 1:** Final core outcome set (COS) by workflow stage.

COS outcome	Pre‐treatment				During treatment		Post‐treatment	
	Patient selection	Diagnostic tools	Treatment planning	Pre‐op*	Treatment procedure	Management of complications during treatment procedure	Maintenance	Management of complications during maintenance
*Patient‐Reported Outcome (PRO)*								
Aesthetic satisfaction				X				
Chewing function/comfort/discomfort								
Complications during treatment/maintenance								X
Ease of cleaning/oral hygiene efficacy							X	
Pain					X			
Patient overall satisfaction with treatment							X	
Patient‐reported complaints								
Prosthesis retention/stability								
Quality of life (OHRQoL)								
Speech/phonetics/pronunciation function								
*Objective Clinician‐Reported Outcome (ClinRO)*								
Biological complications							X	
History of patient compliance	X			X				
Implant failure								X
Implant primary stability					X			
Implant success							X	
Implant survival							X	
Mechanical/technical complications							X	X
Peri‐implant health (implant level)							X	
Peri‐implant health (patient level)							X	
Peri‐implant mucositis							X	
Peri‐implant suppuration							X	X
Peri‐implantitis							X	X
Plaque index/Oral hygiene				X			X	
Postoperative complications						X		
Presence of keratinized mucosa								
Prosthesis failure								X
Prosthesis success							X	
Prosthetic complications							X	X
Radiographic marginal bone level							X	
Radiographic marginal bone loss							X	
Surgical/intraoperative complications								
Width of keratinized mucosa								
*Subjective Clinician‐Reported Outcome (ClinRO)*								
Clinician's treatment success							X	
Prosthodontic maintenance events/complications							X	X

*Note:* “X” indicates outcomes that were rated as relevant by at least 75% of participants for a given stage. In the pooled analysis, an outcome was considered relevant to the pre‐treatment phase (Pre‐op) if it was selected in at least one of the pre‐treatment workflow stages. Pre‐op* = Patient selection + Diagnostic tools + Treatment planning.

Abbreviation: OHRQoL: oral health‐related quality of life.

## 4 Discussion

Across eight systematic reviews, outcomes and measurement instruments were highly heterogeneous. Implant or prosthesis survival and success, marginal bone level change, and biological or mechanical complications were consistently reported, whereas patient‐reported outcomes (PROs; e.g., chewing comfort, speech, aesthetics, oral health–related quality of life), and timing of assessments were reported inconsistently and infrequently. Using a three‐round, anonymous Delphi process with high response rates (87.6%, 95.2%, 92.4%), a COS for rehabilitation of the edentulous maxilla was identified, comprising 10 PROs, 22 objective ClinROs, and 2 subjective ClinROs. Each outcome was mapped to specific stages of care to guide when assessments are most relevant.

The emphasis on PROs highlights a growing recognition that patients' perspectives are central to defining treatment success. Outcomes such as chewing function, speech, aesthetic satisfaction, and oral‐health–related quality of life were consistently rated as critically important, aligning with evidence that patient satisfaction and function are key determinants of treatment acceptance and long‐term success (Abou‐Ayash et al. 2023; Lang and Zitzmann 2012; Tonetti et al. 2025; Tonetti, Sanz, et al. 2023; Yao et al. 2018). Additionally, outcomes such as pain, ease of cleaning, and overall satisfaction emphasize the importance of capturing patients' experiences beyond the surgical phase and into daily function and maintenance.

Objective ClinROs, such as implant survival, prosthesis success, biological complications, and radiographic marginal bone loss, were also prioritized. These remain essential endpoints of clinical success and are well established in the literature as predictors of long‐term outcomes (Tonetti, Heitz‐Mayfield, et al. 2023). The inclusion of peri‐implant health at both the implant and patient levels reflects increasing evidence supporting their role in maintaining peri‐implant tissue stability and preventing disease progression (Herrera et al. 2023). Subjective ClinROs, including clinician‐perceived treatment success and prosthodontic maintenance complexity, were also incorporated. While inherently more variable, these measures provide important context regarding the feasibility, resource requirements and real‐world considerations of care delivery.

The Delphi process mapped each outcome to specific stages of care, ranging from patient selection and treatment planning to post‐treatment maintenance. When these stages were grouped into pre‐, within‐, and post‐treatment ones, only a limited number of outcomes were identified as important across multiple phases. For example, plaque index/oral hygiene was considered relevant both before and after treatment, while patient compliance was rated as important before and during treatment.

A total of 21 COS outcomes were identified as relevant to the maintenance phase, either for routine follow‐up or for managing complications after treatment completion, with limited emphasis on baseline or within‐treatment assessments. This distribution limits the ability to capture changes over time, as outcomes recorded only after treatment lack the context provided by earlier measurements (Young et al. 2019).

The GCCG's focus on rehabilitation of the fully edentulous maxilla may also explain why certain outcomes, such as clinical parameters, were underrepresented in pre‐ and within‐treatment stages. This could reflect participants' interpretation of peri‐implant disease. Indeed, the timing and perceived relevance of these outcomes are likely influenced by factors such as opposing dentition status and prior implant interventions. Overall, this stage‐specific framework enhances the clinical utility of the COS by identifying not only *what* should be measured but also *when* these measurements should occur across the continuum of care.

Prior initiatives to standardize outcome reporting in implant dentistry have laid an essential foundation (Sanz et al. 2023; Tonetti, Heitz‐Mayfield, et al. 2023). However, implant dentistry is a complex and multidisciplinary field involving prosthodontists, periodontists, oral and maxillofacial surgeons and general practitioners. It also includes a wide range of clinical scenarios, such as the rehabilitation of partially or fully edentulous arches in the maxilla, mandible or both. Given this diversity, earlier COS initiatives (Tonetti, Heitz‐Mayfield, et al. 2023) while essential, require further refinement to accommodate the variety of treatment contexts and clinical perspectives.

Importantly, the use of a COS does not imply that outcome reporting in clinical trials is limited to the core set alone. Rather, it establishes a minimum set of outcomes that should be consistently measured and reported across studies, while allowing researchers the flexibility to include additional outcomes relevant to their specific objectives (Williamson et al. 2017). Moreover, COS frameworks are expected to evolve over time in response to advances in research, technology and clinical practice. The GCCG participants noted that, for some reporting items considered for inclusion, the supporting empirical evidence, available instruments and methodological guidance remain limited. These items were most likely deferred until their relevance and application are more clearly understood. The current effort builds on existing foundations by introducing a more comprehensive and context‐sensitive COS, better aligned with the complexities of modern implant dentistry.

A major strength of this initiative lies in its broad, multidisciplinary engagement and the use of a rigorous Delphi consensus process. High response rates across three rounds, ranging from 87.6% to 95.2%, underscore the level of interest and global commitment to standardizing outcome reporting in this field. The resulting COS reflects a comprehensive integration of PROs, objective ClinROs, and subjective ClinROs, acknowledging the multifactorial nature of successful rehabilitation in patients with edentulous maxillae.

This study also has limitations. First, although a COS for the edentulous maxilla was identified, assessing its quality remains challenging (Kirkham et al. 2017). Ideally, an optimal COS is one that is adopted in practice and ultimately contributes to better patient outcomes (Williamson et al. 2017). However, such benefits occur long after its development, making it impossible to evaluate them based solely on the current study (Williamson et al. 2017). Second, while patients and cross‐disciplinary experts contributed to proposing relevant outcomes, unlike the ID‐COSM initiative (Sanz et al. 2023), they did not directly participate in the Delphi consensus process. We plan formal patient partner involvement in subsequent phases (e.g., instrument selection, plain‐language labels, and timing of assessment). Third, although outcome constructs were selected, the measurement tool for certain outcomes, particularly some PROMs, remains undefined due to the lack of agreed‐upon measurement instruments in the literature (Powers 3rd et al. 2017; Prinsen et al. 2016). The development of a validated PROM requires a rigorous, multistep process that incorporates both qualitative and quantitative methods, which is beyond the scope of this study (Calvert et al. 2021; Rothrock et al. 2011). Fourth, achieving consensus on pre‐treatment‐ related outcomes proved more difficult, likely due to variation in diagnostic protocols, clinical workflows and professional roles across different regions and specialties. This variability underscores the need for future work to harmonize diagnostic criteria and risk stratification tools. Although many outcomes were identified as relevant in the maintenance phase, determining the optimal timing and frequency of their assessment could be an important focus for future research to enhance their application in clinical practice and research (Young et al. 2019). Indeed, variability in the timing of outcome reporting contributes to increased heterogeneity and compromises the comparability of study findings (Messias et al. 2023).

Additionally, some ClinROs encompassed overlapping concepts, a mixture of signs, symptoms, and diagnoses. While “ClinROs” served as an umbrella term for simplicity this may cause ambiguity among clinicians. Interestingly, participants identified diagnostic terms (e.g., peri‐implant mucositis, peri‐implantitis) but not clinical parameters such as probing depth or bleeding on probing, possibly reflecting ongoing debate regarding the value of routine probing in stable cases without complications during supportive peri‐implant therapy (Coli et al. 2017; Coli and Sennerby 2019).

Despite efforts to ensure broad geographical participation, most invitees were from North America, South America, and Europe rather than from Asia, Africa and Oceania. This imbalance may have biased the findings toward the practices and priorities of overrepresented regions, potentially limiting the global generalizability, particularly for settings with different healthcare systems, resources, and patient needs.

For the first time, an initiative identifies a COS that captures both patient‐ and clinician‐relevant outcomes for the treatment of the edentulous maxilla. This COS development represents a significant advancement toward improving the quality, comparability and clinical relevance of research in implant dentistry. Despite a growing number of studies in this area, inconsistencies in outcome reporting have limited the ability to synthesize findings across trials, undermining efforts to generate robust evidence and inform clinical guidelines. Through an internationally coordinated effort grounded in established methodology from the COMET Initiative (Williamson et al. 2017), the 1st GCCG addressed this gap by establishing a standardized set of outcomes that reflect both clinical priorities and patient perspectives. The COS should be viewed as a living framework, periodically updated to incorporate new evidence and emerging perspectives, ensuring it remains both relevant and comprehensive.

This consensus‐based COS provides a foundational framework for future clinical trials and systematic reviews in implant dentistry focused on the edentulous maxilla. By aligning outcome reporting with stakeholder priorities and clinical relevance, the COS offers a pathway toward more transparent, comparable, and patient‐centered research and will inform evidence‐based clinical guidelines globally. Uptake will be promoted through dissemination across dental associations, conferences, and professional networks.

## 5 Conclusions

The proposed COS for studies on the rehabilitation of the edentulous maxilla in implant dentistry was developed using the COMET methodology. Although patients' perspectives were incorporated during the preliminary survey phase, they did not directly participate in the Delphi, highlighting an opportunity for future research to further enhance patient involvement in COS development. The widespread implementation of this COS is expected to improve the consistency of outcome reporting and facilitate comparisons of the effectiveness of current and emerging interventions.

## Author Contributions

Conceptualization: I.K., H.‐L.W., F.S.; Methodology: G.B., I.K., F.J.S.; Software: G.B., F.J.S.; Validation: G.B., F.J.S.; Formal analysis: G.B., F.J.S.; Investigation: G.B., F.J.S.; Resources: H.‐L.W., F.S., R.E.J.; Data curation: G.B., G.‐H.L., I.K., A.C.‐L., F.J.S.; Writing – original draft: G.B., F.J.S.; Writing – review and editing: G.B., G.‐H.L., I.K., A.C.‐L., R.E.J, H.‐L.W., F.S., F.J.S.; Visualization: G.B., F.J.S.; Supervision: I.K., R.E.J., H.‐L.W., F.S.; Project administration: R.E.J., H.‐L.W., F.S.

## Funding

The GCCG was funded by grants from the European Association for Osseointegration, the International Team for Implantology, and the Osteology Foundation.

## Ethics Statement

The authors have nothing to report.

## Conflicts of Interest

All delegates disclosed secondary interests using the standardized ICMJE disclosure form. Potential conflicts of interest (CoIs) were actively managed in accordance with Guidelines International Network (GIN) principles.

## Supporting information


**Appendix S1:** clr70075‐sup‐0001‐AppendixS1.pdf.

**Appendix S2:** clr70075‐sup‐0002‐AppendixS2.pdf.

**Appendix S3:** clr70075‐sup‐0003‐AppendixS3.pdf.

**Figure S1:** Medians and interquartile ranges (IQRs) of patient‐reported outcomes (PROs) scored in the first‐round Delphi survey (92 respondents).
**Figure S2:** Medians and interquartile ranges (IQRs) of objective clinician‐reported outcomes (ClinROs) scored in the first‐round Delphi survey (90 respondents).
**Figure S3:** Medians and interquartile ranges (IQRs) of subjective clinician‐reported outcomes (ClinROs) scored in the first‐round Delphi survey (88 respondents).
**Figure S4:** Medians and interquartile ranges (IQRs) of patient‐reported outcomes (PROs) scored in the second‐round Delphi survey (100 respondents).
**Figure S5:** Medians and interquartile ranges (IQRs) of objective clinician‐reported outcomes (ClinROs) scored in the second‐round Delphi survey (99 respondents): Implant performance; Implant‐supported prosthesis performance; Surgical domain; Peri‐implant tissue health.
**Figure S6:** Medians and interquartile ranges (IQRs) of subjective clinician‐reported outcomes (ClinROs) scored in the second‐round Delphi survey (99 respondents).

**Table S1:** List of patient‐reported outcomes (PROs) of the first‐round Delphi survey (92 respondents). Data are expressed as percentages (%).
**Table S2:** List of objective clinician‐reported outcomes (ClinROs) of the first‐round Delphi survey (90 respondents). Data are expressed as percentages (%).
**Table S3:** List of subjective clinician‐reported outcomes (ClinROs) of the first‐round Delphi survey (88 respondents). Data are expressed as percentages (%).
**Table S4:** List of patient‐reported outcomes (PROs) of the second‐round Delphi survey (100 respondents). Data are expressed as percentages (%).
**Table S5:** List of objective clinician‐reported outcomes (ClinROs) of the second‐round Delphi survey (99 respondents). Data are expressed as percentages (%).
**Table S6:** List of subjective clinician‐reported outcomes (ClinROs) of the second‐round Delphi survey (99 respondents). Data are expressed as percentages (%).
**Table S7:** List of patient‐reported outcomes (PROs) of the third‐round Delphi survey (97 respondents). Data are expressed as percentages (%).
**Table S8:** List of objective clinician‐reported outcomes (ClinROs) of the third‐round Delphi survey (97 respondents). Data are expressed as percentages (%).
**Table S9:** List of subjective clinician‐reported outcomes (ClinROs) of the third‐round Delphi survey (96 respondents). Data are expressed as percentages (%).

## Data Availability

The data that support the findings of this study are available from the corresponding authors upon reasonable request.
