# Supplementary material for: 1st Global Consensus for Clinical Guidelines: Identifying a Core Outcome Set for Implant Dentistry in Edentulous Maxilla Rehabilitation
Source: Clin Oral Implants Res. 2026 Feb 24;37(Suppl 30):S108–20. doi: 10.1111/clr.70075 (PMC12930137; doi:10.1111/clr.70075)
Supplement: Supplementary file 1 — Appendix S1: clr70075‐sup‐0001‐AppendixS1.pdf. [file CLR-37-S108-s001.pdf]

* 1. Please rate, on a scale from 1 to 9, the importance of the following **patient-reported outcomes** in the context of rehabilitating the edentulous maxilla. All the outcomes have to be rated.

	1 of **least** importance	2	3	4	5	6	7	8	9 of **most** importance
Aesthetic improvement from patient's perspective	◯	◯	◯	◯	◯	◯	◯	◯	◯
Aesthetics (satisfaction)	◯	◯	◯	◯	◯	◯	◯	◯	◯
Articulation profile	◯	◯	◯	◯	◯	◯	◯	◯	◯
Chewing function	◯	◯	◯	◯	◯	◯	◯	◯	◯
Comfort	◯	◯	◯	◯	◯	◯	◯	◯	◯
Comfort/chewing discomfort	◯	◯	◯	◯	◯	◯	◯	◯	◯
Complications during maintenance phase	◯	◯	◯	◯	◯	◯	◯	◯	◯
Complications during treatment procedure	◯	◯	◯	◯	◯	◯	◯	◯	◯
Days of impaired activity	◯	◯	◯	◯	◯	◯	◯	◯	◯
Discomfort with the surgery	◯	◯	◯	◯	◯	◯	◯	◯	◯
Duration of the surgery	◯	◯	◯	◯	◯	◯	◯	◯	◯
Ease of cleaning	◯	◯	◯	◯	◯	◯	◯	◯	◯
Feeling of "being my own teeth"	◯	◯	◯	◯	◯	◯	◯	◯	◯
Functional limitations	◯	◯	◯	◯	◯	◯	◯	◯	◯
Handicap	◯	◯	◯	◯	◯	◯	◯	◯	◯
Immediate post-surgery prosthesis	◯	◯	◯	◯	◯	◯	◯	◯	◯
Invasiveness of the procedure	◯	◯	◯	◯	◯	◯	◯	◯	◯
Likelihood to repeat surgery	◯	◯	◯	◯	◯	◯	◯	◯	◯
Loading protocol	◯	◯	◯	◯	◯	◯	◯	◯	◯
Number of surgeries	◯	◯	◯	◯	◯	◯	◯	◯	◯
Occlusion	◯	◯	◯	◯	◯	◯	◯	◯	◯

Oromyofunction									
Pain									
Patient overall satisfaction									
Patient-reported complaints									
Physical disability									
Physical discomfort									
Postoperative swelling									
Prosthesis retention/stability									
Psychological disability									
Psychological discomfort									
Psychosocial impact (comfort)									
Quality of life (e.g. OHIP-14)									
Self-esteem									
Social disability									
Speech/phonetics/pronunciation function									
Treatment costs/cost-effectiveness									
Treatment time/overall duration of the treatment									
Type of definitive prosthesis (i.e. fixed vs removable)									
Whether the patient would recommend the treatment									

Global Consensus for Clinical Guidelines

* 2. Please rate, on a scale from 1 to 9, the importance of the following **objective clinician-reported outcome measures** in the context of rehabilitating the edentulous maxilla. All the outcomes have to be rated.

	1 of **least** importance	2	3	4	5	6	7	8	9 of **most** importance
Articulation disorder	○	○	○	○	○	○	○	○	○
Biological complications	○	○	○	○	○	○	○	○	○
Bleeding on probing (BoP)	○	○	○	○	○	○	○	○	○
Bleeding scores	○	○	○	○	○	○	○	○	○
Bone quality	○	○	○	○	○	○	○	○	○
Bone quantity	○	○	○	○	○	○	○	○	○
CBCT changes	○	○	○	○	○	○	○	○	○
Clinical attachment level (CAL)	○	○	○	○	○	○	○	○	○
Clinical bone gain	○	○	○	○	○	○	○	○	○
Clinical complications	○	○	○	○	○	○	○	○	○
Clinical examination	○	○	○	○	○	○	○	○	○
Complications	○	○	○	○	○	○	○	○	○
CT matching	○	○	○	○	○	○	○	○	○
Denture survival	○	○	○	○	○	○	○	○	○
Duration of surgery	○	○	○	○	○	○	○	○	○
EMG activity of masseters and temporalis	○	○	○	○	○	○	○	○	○
Gingival index	○	○	○	○	○	○	○	○	○
Histological outcomes	○	○	○	○	○	○	○	○	○
Hospitalization period	○	○	○	○	○	○	○	○	○

Implant complications									
Implant failure									
Implant insertion torque									
Implant mobility									
Implant primary stability									
Implant reverse torque									
Implant stability									
Implant success									
Implant survival									
Implant-Quality Scale									
Intraoperative complications									
Keratinized tissue width									
Marginal bone level									
Marginal bone loss									
Masticatory performance									
Maxillary sinus volume changes									
Mechanical complications									
Morbidity and surgery-related complications									
Number of treatment sessions									
Occlusal force/area by digital devices									
Occlusal forces									
Peri-implant bone remodeling									
Peri-implant diseases									
Peri-implant health									
Peri-implant mucositis									
Peri-Implant soft tissue conditions									
Peri-implant suppuration									
Peri-implantitis									
Periodontal parameters									

Plaque index	◯	◯	◯	◯	◯	◯	◯	◯	◯
Pocket depth	◯	◯	◯	◯	◯	◯	◯	◯	◯
Postoperative complications	◯	◯	◯	◯	◯	◯	◯	◯	◯
Postoperative swelling	◯	◯	◯	◯	◯	◯	◯	◯	◯
Prosthesis failure (repeated overdenture fractures requiring metallic reinforcement)	◯	◯	◯	◯	◯	◯	◯	◯	◯
Prosthesis failures	◯	◯	◯	◯	◯	◯	◯	◯	◯
Prosthesis success	◯	◯	◯	◯	◯	◯	◯	◯	◯
Prosthetic complications	◯	◯	◯	◯	◯	◯	◯	◯	◯
Prosthodontic maintenance	◯	◯	◯	◯	◯	◯	◯	◯	◯
Prosthodontic outcomes	◯	◯	◯	◯	◯	◯	◯	◯	◯
Provisional complications	◯	◯	◯	◯	◯	◯	◯	◯	◯
Radiographic evaluation	◯	◯	◯	◯	◯	◯	◯	◯	◯
Radiographic measurements	◯	◯	◯	◯	◯	◯	◯	◯	◯
Recession	◯	◯	◯	◯	◯	◯	◯	◯	◯
RFA implant stability	◯	◯	◯	◯	◯	◯	◯	◯	◯
Ridge height measurement	◯	◯	◯	◯	◯	◯	◯	◯	◯
Ridge width measurement	◯	◯	◯	◯	◯	◯	◯	◯	◯
Surgical complications	◯	◯	◯	◯	◯	◯	◯	◯	◯
Technical complications	◯	◯	◯	◯	◯	◯	◯	◯	◯
Time to function	◯	◯	◯	◯	◯	◯	◯	◯	◯
Vertical bone height on CBCT	◯	◯	◯	◯	◯	◯	◯	◯	◯

Global Consensus for Clinical Guidelines

* 3. Please rate, on a scale from 1 to 9, the importance of the following **subjective clinician-reported outcome measures** (clinicians' perception) in the context of rehabilitating the edentulous maxilla. All the outcomes have to be rated.

	1 of **least** importance	2	3	4	5	6	7	8	9 of **most** importance
Aesthetic satisfaction (Abutment visibility)	○	○	○	○	○	○	○	○	○
Aesthetic satisfaction (Appearance)	○	○	○	○	○	○	○	○	○
Aesthetic satisfaction (Papilla)	○	○	○	○	○	○	○	○	○
Aesthetic satisfaction (Pink ceramics)	○	○	○	○	○	○	○	○	○
Aesthetic satisfaction (Restorations)	○	○	○	○	○	○	○	○	○
Aesthetic satisfaction (Speech)	○	○	○	○	○	○	○	○	○
Clinician satisfaction of aesthetics	○	○	○	○	○	○	○	○	○
Clinician's treatment success	○	○	○	○	○	○	○	○	○
Prosthodontic maintenance events	○	○	○	○	○	○	○	○	○
Surgery difficulty	○	○	○	○	○	○	○	○	○

Global Consensus for Clinical Guidelines

4. Do you believe there are additional **patient-reported outcomes** relevant to the rehabilitation of the edentulous maxilla that were not included in the current list? If so, you have the possibility to suggest below up to five outcomes (one per box).

1

2

3

4

5

5. Do you believe there are additional **clinician-reported outcome measures** relevant to the rehabilitation of the edentulous maxilla that were not included in the current list? If so, you have the possibility to suggest below up to five outcomes (one per box).

1

2

3

4

5

Please remember to click the '**Done**' button to submit your response!
