# Supplementary material for: 1st Global Consensus for Clinical Guidelines: Identifying a Core Outcome Set for Implant Dentistry in Edentulous Maxilla Rehabilitation
Source: Clin Oral Implants Res. 2026 Feb 24;37(Suppl 30):S108–20. doi: 10.1111/clr.70075 (PMC12930137; doi:10.1111/clr.70075)
Supplement: Supplementary file 2 — Appendix S2: clr70075‐sup‐0002‐AppendixS2.pdf. [file CLR-37-S108-s002.pdf]

Global Consensus for Clinical Guidelines

* 1. Please rate, on a scale from 1 to 9, the importance of the following **patient-reported outcomes** in the context of rehabilitating the edentulous maxilla. All the outcomes have to be rated.

	1 of **least** importance	2	3	4	5	6	7	8	9 of **most** importance
**"Achiness" as day progresses** - Incremental discomfort over the course of the day									
**"Achiness": upon awakening** - Discomfort or soreness felt immediately after waking up									
**Aesthetic satisfaction** - Aesthetic improvement from patient’s perspective using Visual Analog Scale (VAS) (0-100) or a Numeric Rating scale (NRS) (0-10)									
**Chewing function/comfort/discomfort** - Using VAS (0-100) or a NRS (0-10)									
**Complaint handling (who and how)** - Patient perception of the responsiveness and quality of how their concerns or complaints are addressed									
**Complications treatment/maintenance** - Any report that negatively affects the patient's well-being that can be reported by themselves									
**Cost of repairs/maintenance** - Patient perception of the financial burden of ongoing treatment upkeep									
**Days of impaired activity or days of recovery (invasiveness of the procedure)** - As an indirect measure to evaluate invasiveness of the procedure									
**Decision Regret Scale (DRS) - Brehaut et al. (2003) -** A 5- item scale assessing different health care decisions. Scores are									

converted to a 100-point scale, with higher scores corresponding to higher levels of regret. The instrument has been used extensively for oncologic screening and treatment decisions	○	○	○	○	○	○	○	○	○
**Discomfort with the surgery** - e.g. using VAS (0-100) or a NRS (0-10)	○	○	○	○	○	○	○	○	○
**Discussion of a non-implant alternative therapy** - Patient perception of receiving information on treatment alternatives to implants	○	○	○	○	○	○	○	○	○
**Ease of cleaning /oral hygiene efficacy** - e.g. using VAS (0-100) or a NRS (0-10) asking “How difficult is it for you to clean your implant-supported prosthesis?’	○	○	○	○	○	○	○	○	○
**Feeling of “being my own teeth”** - Using VAS (0-100) or a NRS (0-10)	○	○	○	○	○	○	○	○	○
**Gag reflex/desire for no palatal coverage** - Patient reported gag reflex to avoid palate coverage in the prosthesis	○	○	○	○	○	○	○	○	○
**Immediate post-surgery prosthesis** - Preference of the patient regarding the timing of provisional	○	○	○	○	○	○	○	○	○
**Increased headache frequency** - Increased headache frequency reported by the patient after treatment	○	○	○	○	○	○	○	○	○
**Influence on TMJ complaints** - Patient perception of changes in jaw joint pain or dysfunction following treatment	○	○	○	○	○	○	○	○	○
**Likelihood to repeat surgery** - e.g. using VAS (0-100) or a NRS (0-10) asking “How likely would you repeat this surgery?"	○	○	○	○	○	○	○	○	○
**Lip esthetics (Lip support)** - Patient perception of how well the treatment enhances or maintains the appearance and support of the lips	○	○	○	○	○	○	○	○	○
**Loading protocol preference** - Patient preference of loading protocol	○	○	○	○	○	○	○	○	○
**Malodor** - Patient self-reported perception of unpleasant oral odor	○	○	○	○	○	○	○	○	○
**Mucosal cheek or tongue biting** - Patient self-reported experience of accidental biting or injury to cheek or tongue	○	○	○	○	○	○	○	○	○
**Number of appointments**	○	○	○	○	○	○	○	○	○
**Number of surgeries**	○	○	○	○	○	○	○	○	○

Pain - e.g. using VAS (0-100) or NRS (0-10) filled out by the patient after surgery

Passive fit for immediate function - Patient perception of precise adaptation of the restoration to oral tissues allowing for immediate use

Patient overall satisfaction with treatment - For example, by using VAS (0-100) or a NRS (0-10)

Patient perceived health status - e.g. using the SF-36 questionnaire designed to capture patients' perceptions of their own health and well-being across eight dimensions: physical functioning, role limitations due to physical health, bodily pain, general health, vitality, social functioning, role limitations due to emotional problems, and mental health

Patient-reported complaints - Concerns directly expressed by the patient

Postoperative swelling - e.g. using VAS (0-100) or NRS (0-10) filled out by the patient after surgery

Prosthesis retention/stability - e.g. using VAS (0-100) or a NRS (0-10)

Rehabilitation journey's perception of difficulty to them - As perceived by the patient e.g. using VAS (0-100) or a NRS (0-10)

Quality of life (e.g. OHIP-14) - Oral-health related quality of life using OHIP-14, which evaluates 7 domains; Functional limitation, Physical pain, Psychological discomfort: Physical disability, Psychological disability, Social disability and Handicap

Shared decision making - Patient perception of involvement in treatment choices

Speech/phonetics/pronunciation function - Using VAS (0-100) or a NRS (0-10)

Treatment costs/cost-effectiveness - Patient perception of whether the treatment’s benefits justify the financial burden

Treatment time/overall duration of the treatment - Duration of treatment (months from beginning to end and number of appointments)

Treatment-related anxiety /

**stress** - Patient self-reported emotional response of worry or stress associated with the treatment	○	○	○	○	○	○	○	○	○
**Type of definitive prosthesis (i.e. fixed vs removable)** - Patient preference of rehabilitation	○	○	○	○	○	○	○	○	○
**What is the maximum number of surgeries you feel you could tolerate for this treatment?** - As perceived by the patient	○	○	○	○	○	○	○	○	○
**Whether the patient would recommend the treatment** - e.g. using VAS (0-100) or a NRS (0-10) asking “How likely would you recommend this treatment	○	○	○	○	○	○	○	○	○

Global Consensus for Clinical Guidelines

* 2. Please rate, on a scale from 1 to 9, the importance of the following **objective clinician-reported outcome measures** in the context of rehabilitating the edentulous maxilla (**category: Implant performance**). All the outcomes have to be rated.

	1 of **least** importance	2	3	4	5	6	7	8	9 of **most** importance
**Articulation disorder** - Speech disorders characterized by impaired ability to produce certain sounds	○	○	○	○	○	○	○	○	○
**Clinical attachment level** - Clinical attachment level of opposing arch if there are teeth present	○	○	○	○	○	○	○	○	○
**Denture survival** - No need to be replaced by an alternative prosthesis	○	○	○	○	○	○	○	○	○
**EMG activity of masseters and temporalis** - EMG (electromyographic) activity of the masseter and temporalis muscles	○	○	○	○	○	○	○	○	○
**Implant failure** - Implant does not comply with survival / success criteria. Usually associated with mobility, infection, fracture, etc.	○	○	○	○	○	○	○	○	○
**Implant success** - Implant in the patient's mouth fulfilling any pre-established set of success criteria	○	○	○	○	○	○	○	○	○
**Implant survival** - An implant present in the patient’s mouth, irrespective of its peri-implant health condition or functional	○	○	○	○	○	○	○	○	○

status of the prosthesis.									
**Masticatory performance** - Chewing efficiency assessed with a two-color wax tablet chewed 20 times (Mixing Ability Index)	○	○	○	○	○	○	○	○	○
**Mechanical/technical complications** - Mechanical and technical complications of prosthesis or dental implants (e.g. framework fractures, wearing out, screw loosening, chipping, abutment fracture, implant fracture etc.)	○	○	○	○	○	○	○	○	○
**Occlusal force/occlusal area by digital devices** - Distribution of occlusal forces and area analyzed using digital devices	○	○	○	○	○	○	○	○	○
**Occlusal forces** - Distribution of occlusal forces analyzed using a pressure-sensitive film	○	○	○	○	○	○	○	○	○
**Periodontal parameters** - Parameters of the opposing arch if teeth are present (e.g. PD, BOP, CAL)	○	○	○	○	○	○	○	○	○

* 3. Please rate, on a scale from 1 to 9, the importance of the following **objective clinician-reported outcome measures** in the context of rehabilitating the edentulous maxilla (**category: Implant-supported prosthesis performance**). All the outcomes have to be rated.

	1 of **least** importance	2	3	4	5	6	7	8	9 of **most** importance
**Plaque index / Oral Hygiene** - Full mouth plaque index (site presence yes/ total number of sites)	◯	◯	◯	◯	◯	◯	◯	◯	◯
**Prosthesis failure** - Prosthesis functionally compromised or lost for any reason	◯	◯	◯	◯	◯	◯	◯	◯	◯
**Prosthesis success** - Prosthesis is stable, in function and complication-free	◯	◯	◯	◯	◯	◯	◯	◯	◯
**Prosthetic complications** - Mechanical/technical complications at the affecting the restoration	◯	◯	◯	◯	◯	◯	◯	◯	◯
**Prosthodontic maintenance appointments needed** - Frequency of prosthodontic maintenance (e.g. denture relining, bar clip or O-ring change, etc)	◯	◯	◯	◯	◯	◯	◯	◯	◯
**Provisional complications** - Complications at the provisional prosthesis (e.g. fracture of the provisional prosthesis)	◯	◯	◯	◯	◯	◯	◯	◯	◯

* 4. Please rate, on a scale from 1 to 9, the importance of the following **objective clinician-reported outcome measures** in the context of rehabilitating the edentulous maxilla (**category: Surgical domain**). All the outcomes have to be rated.

	1 of **least** importance	2	3	4	5	6	7	8	9 of **most** importance
**Accuracy of implant placement via CT matching** - Accuracy of surgical implant placement assessed via CT matching	◯	◯	◯	◯	◯	◯	◯	◯	◯
**Bone quality** - Index Leckhom & Zarb (bone types I-V)	◯	◯	◯	◯	◯	◯	◯	◯	◯

**Bone quantity** - Index Leckhom & Zarb (bone types A-E)	○	○	○	○	○	○	○	○	○
**CBCT Linear and/or volumetric changes** - Linear and/or volumetric bone changes overtime in subsequent CBCTs	○	○	○	○	○	○	○	○	○
**Clinical bone gain** - Direct clinical bone dimensions using a caliper at the baseline and the re-entry surgery for implant placement	○	○	○	○	○	○	○	○	○
**Duration of surgery** - Total length of surgery	○	○	○	○	○	○	○	○	○
**Histological outcomes** - Histological evaluation through bone biopsies	○	○	○	○	○	○	○	○	○
**Hospitalization period** - Total days hospitalized due to surgery	○	○	○	○	○	○	○	○	○
**Implant insertion torque** - Measured with the implant engine when introducing the implant (expressed in N-cm)	○	○	○	○	○	○	○	○	○
**Implant primary stability** - Implant stability at implant placement	○	○	○	○	○	○	○	○	○
**Implant reverse torque** - Reverse-torque testing for verifying osseointegration	○	○	○	○	○	○	○	○	○
**Implant stability/mobility** - Determination of implant stability/mobility (either manually thorough torque control gauge or by RFA)	○	○	○	○	○	○	○	○	○
**Maxillary sinus height/volume changes** - Changes between two different time points using CBCT	○	○	○	○	○	○	○	○	○
**Number of treatment sessions** - Number of appointments needed to complete the treatment	○	○	○	○	○	○	○	○	○
**Peri-implant bone remodeling via CBCT** - Mean bone changes occurring after implant placement and follow-up time point by calculating the distance from the mesial and distal cervical bone levels to the apex of the implant	○	○	○	○	○	○	○	○	○

**Postoperative complications** - Complications that occur after surgical procedures (e.g. implant placement or sinus lift), such as wound dehiscence, bleeding, hematoma, pain, infection, swelling, trismus									
**Postoperative swelling** - Postoperative swelling rated by the clinician using a numerical rating scale									
**Presence of keratinized mucosa** - Presence/absence of a minimum amount of keratinized mucosa (>0mm)									
**Radiographic marginal bone level** - Linear measurements between the most coronal implant-bone contact and the implant platform/shoulder.									
**Radiographic marginal bone loss** - Bone loss occurring between two peri-implant bone level measurements taken at two different time intervals									
**RFA Implant stability** - Determination of implant stability by Resonance Frequency Analysis (RFA)									
**Ridge height changes (3D)** - Vertical bone height changes from a fixed anatomical reference measured using CBCT									
**Ridge width changes (3D)** - Horizontal dimension changes of the alveolar crest measured using CBCT									
**Surgical/intraoperative complications** - Complications that occur during surgical procedures (e.g. implant placement or sinus lift), such as intraoperative bleeding, sinus perforation, or nerve injury									
**Time to function** -									

Number of days from the initial surgical intervention to the delivery of the implant-supported provisional prosthesis	◯	◯	◯	◯	◯	◯	◯	◯	◯
**Width of keratinized mucosa** - Peri-implant width of the keratinized mucosa	◯	◯	◯	◯	◯	◯	◯	◯	◯

* 5. Please rate, on a scale from 1 to 9, the importance of the following **objective clinician-reported outcome measures** in the context of rehabilitating the edentulous maxilla (**category: Peri-implant tissue health**). All the outcomes have to be rated.

	1 of **least** importance	2	3	4	5	6	7	8	9 of **most** importance
**Biological complications** - Mucositis, peri-implantitis, lack of keratinized mucosa, implant mobility etc...	◯	◯	◯	◯	◯	◯	◯	◯	◯
**Bleeding on probing** - Determination of bleeding on gentle probing (site evaluation bleeding yes/no)	◯	◯	◯	◯	◯	◯	◯	◯	◯
**History of patient compliance** - Compliers or erratic patients e.g. fewer than 2 appointments per year	◯	◯	◯	◯	◯	◯	◯	◯	◯
**Peri-implant Health (implant level)** - No BOP and no bone loss	◯	◯	◯	◯	◯	◯	◯	◯	◯
**Peri-implant Health (patient level)** - No implant in patient's mouth depicting BOP and no bone loss	◯	◯	◯	◯	◯	◯	◯	◯	◯
**Peri-implant mucositis** - BOP with or without suppuration and deep PD; no bone loss	◯	◯	◯	◯	◯	◯	◯	◯	◯
**Peri-implant suppuration** - Determination of suppuration upon gentle probing (site evaluation suppuration yes/no)	◯	◯	◯	◯	◯	◯	◯	◯	◯
**Peri-implantitis** - BOP and/or suppuration, with or without deep PD; with bone loss	◯	◯	◯	◯	◯	◯	◯	◯	◯
**Pink Esthetic Score (PES)**	◯	◯	◯	◯	◯	◯	◯	◯	◯
**Probing depth** - Determination of the distance between the mucosal margin and the depth of probe penetration	◯	◯	◯	◯	◯	◯	◯	◯	◯

using light probing forces									
**Recession** - Peri-implant marginal recession, mid-facial/ buccal/ soft tissue margin position	○	○	○	○	○	○	○	○	○
**Restorative/interocclusal space**	○	○	○	○	○	○	○	○	○
**United States Public Health Service (USPHS)** - Criteria for Prosthesis Evaluation	○	○	○	○	○	○	○	○	○
**White Esthetic Score (WES)**	○	○	○	○	○	○	○	○	○

* 6. Please rate, on a scale from 1 to 9, the importance of the following **subjective clinician-reported outcome measures** (clinicians' perception) in the context of rehabilitating the edentulous maxilla. All the outcomes have to be rated.

	1 of **least** importance	2	3	4	5	6	7	8	9 of **most** importance
**Aesthetics satisfaction of the clinician** - Aesthetics satisfaction of the clinician based on: (i) abutment visibility, (ii) appearance and presence of the papilla, (iii) aesthetic characteristics of dental restorations, (iv) presence of pink ceramics, (v) external appearance of the patient and (vi) speech via VAS (0-100) or a NRS (0-10)	○	○	○	○	○	○	○	○	○
**Clinician satisfaction of aesthetics** - Clinician satisfaction of aesthetics express for example via VAS	○	○	○	○	○	○	○	○	○
**Clinician's treatment success** - Treatment success assessed by the clinician via VAS (0-100) or a NRS (0-10)	○	○	○	○	○	○	○	○	○
**Complexity of the prosthetic workflow** - As perceived by the clinician	○	○	○	○	○	○	○	○	○
**Digital workflow challenges** - As perceived by the clinician	○	○	○	○	○	○	○	○	○
**Operator's comfort placing short, axial implants vs long implants in tilted manner** - As perceived by the clinician	○	○	○	○	○	○	○	○	○
**Prosthodontic maintenance events/complications** - Perception of the clinician regarding the maintenance of the prosthesis	○	○	○	○	○	○	○	○	○
**Surgery difficulty** - Ease of performing surgical procedures as perceived by the clinician	○	○	○	○	○	○	○	○	○

Global Consensus for Clinical Guidelines

7. Do you believe there are additional **patient-reported outcomes** relevant to the rehabilitation of the edentulous maxilla that were not included in the current list? If so, you have the possibility to suggest below up to five outcomes (one per box).

1

2

3

4

5

8. Do you believe there are additional **clinician-reported outcome measures** relevant to the rehabilitation of the edentulous maxilla that were not included in the current list? If so, you have the possibility to suggest below up to five outcomes (one per box).

1

2

3

4

5

Please remember to click the '**Done**' button to submit your response!
