# Supplementary material for: 1st Global Consensus for Clinical Guidelines: Identifying a Core Outcome Set for Implant Dentistry in Edentulous Maxilla Rehabilitation
Source: Clin Oral Implants Res. 2026 Feb 24;37(Suppl 30):S108–20. doi: 10.1111/clr.70075 (PMC12930137; doi:10.1111/clr.70075)
Supplement: Supplementary file 3 — Appendix S3: clr70075‐sup‐0003‐AppendixS3.pdf. [file CLR-37-S108-s004.pdf]

Global Consensus for Clinical Guidelines

* 1. Please indicate in which stage(s) of the GCCG workflow are relevant for the following **patient-reported outcomes** and **patient-reported outcome measures** in the context of rehabilitating the edentulous maxilla. All outcomes must be assessed, and multiple domains can be selected per outcome.

	Patient selection	Diagnostic tools	Treatment planning	Treatment procedure	Complications during treatment procedure	Maintenance	Complications during maintenance
**Aesthetic satisfaction** - Aesthetic improvement from patient's perspective using Visual Analog Scale (VAS) (0-100) or a Numeric Rating scale (NRS) (0-10)	☐	☐	☐	☐	☐	☐	☐
**Chewing function/comfort/discomfort** - Using VAS (0-100) or a NRS (0-10)	☐	☐	☐	☐	☐	☐	☐
**Complications treatment/maintenance** - Any report that negatively affects the patient's well-being that can be reported by themselves	☐	☐	☐	☐	☐	☐	☐
**Ease of cleaning /oral hygiene efficacy** - e.g. using VAS (0-100) or	☐	☐	☐	☐	☐	☐	☐

a NRS (0-10) asking “How difficult is it for you to clean your implant-supported prosthesis?’	☐	☐	☐	☐	☐	☐	☐
**Pain** - e.g. using VAS (0-100) or NRS (0-10) filled out by the patient after surgery	☐	☐	☐	☐	☐	☐	☐
**Patient overall satisfaction with treatment** - For example, by using VAS (0-100) or a Numeric Rating scale (0-10)	☐	☐	☐	☐	☐	☐	☐
**Patient-reported complaints** - Concerns directly expressed by the patient	☐	☐	☐	☐	☐	☐	☐
**Prosthesis retention/stability** - e.g. using VAS (0-100) or a NRS (0-10)	☐	☐	☐	☐	☐	☐	☐
**Quality of life (e.g. OHIP-14)** - Oral-health related quality of life using OHIP-14, which evaluates 7 domains; Functional limitation, Physical pain, Psychological discomfort: Physical disability, Psychological disability, Social disability and Handicap	☐	☐	☐	☐	☐	☐	☐
**Speech/phonetics/pronunciation function** - Using VAS (0-100) or a NRS (0-10)	☐	☐	☐	☐	☐	☐	☐

* 2. Please indicate in which stage(s) of the GCCG workflow are relevant for the following **objective clinician-reported outcome measures** in the context of rehabilitating the edentulous maxilla (**category: Implant performance**). All outcomes must be assessed, and multiple domains can be selected per outcome.

	Patient selection	Diagnostic tools	Treatment planning	Treatment procedure	Complications during treatment procedure	Maintenance	Complications during maintenance
**Implant failure** - Implant does not comply with survival / success criteria. Usually associated with mobility, infection, fracture, etc.	☐	☐	☐	☐	☐	☐	☐
**Implant success** - Implant in the patient's mouth fulfilling any pre-established set of success criteria	☐	☐	☐	☐	☐	☐	☐
**Implant survival** - An implant present in the patient's mouth, irrespective of its peri-implant health condition or functional status of the prosthesis.	☐	☐	☐	☐	☐	☐	☐
**Mechanical/technical complications** - Mechanical and technical complications of prosthesis or dental implants (e.g. framework fractures, wearing out, screw loosening, chipping, abutment fracture, implant fracture, etc.)	☐	☐	☐	☐	☐	☐	☐

* 3. Please indicate in which stage(s) of the GCCG workflow are relevant for the following **objective clinician-reported outcome measures** in the context of rehabilitating the edentulous maxilla (**category: Implant-supported prosthesis performance**). All outcomes must be assessed, and multiple domains can be selected per outcome.

	Patient selection	Diagnostic tools	Treatment planning	Treatment procedure	Complications during treatment procedure	Maintenance	Complications during maintenance
**Plaque index / Oral Hygiene** - Full mouth plaque index (site presence yes/ total number of sites)	☐	☐	☐	☐	☐	☐	☐
**Prosthesis failure** - Prosthesis functionally compromised or lost for any reason	☐	☐	☐	☐	☐	☐	☐
**Prosthesis success** - Prosthesis is stable, in function and complication-free	☐	☐	☐	☐	☐	☐	☐
**Prosthetic complications** - Mechanical/technical complications at the affecting the restoration	☐	☐	☐	☐	☐	☐	☐

* 4. Please indicate in which stage(s) of the GCCG workflow are relevant for the following **objective clinician-reported outcome measures** in the context of rehabilitating the edentulous maxilla (**category: Surgical domain**). All outcomes must be assessed, and multiple domains can be selected per outcome.

	Patient selection	Diagnostic tools	Treatment planning	Treatment procedure	Complications during treatment procedure	Maintenance	Complications during maintenance
**Implant primary stability** - Implant stability at implant placement	☐	☐	☐	☐	☐	☐	☐
**Postoperative**							

**complications** - Complications that occur after surgical procedures (e.g. implant placement or sinus lift), such as wound dehiscence, bleeding, hematoma, pain, infection, swelling, trismus	☐	☐	☐	☐	☐	☐	☐
**Presence of keratinized mucosa** - Presence/absence of a minimum amount of keratinized mucosa (>0mm)	☐	☐	☐	☐	☐	☐	☐
**Radiographic marginal bone level** - Linear measurements between the most coronal implant-bone contact and the implant platform/shoulder.	☐	☐	☐	☐	☐	☐	☐
**Radiographic marginal bone loss** - Bone loss occurring between two peri-implant bone level measurements taken at two different time intervals	☐	☐	☐	☐	☐	☐	☐
**Surgical/intraoperative complications** - Complications that occur during surgical procedures (e.g. implant placement or sinus lift), such as intraoperative bleeding, sinus perforation, or nerve injury	☐	☐	☐	☐	☐	☐	☐
**Width of keratinized mucosa** - Peri-implant width of the keratinized mucosa	☐	☐	☐	☐	☐	☐	☐

* 5. Please indicate in which stage(s) of the GCCG workflow are relevant for the following **objective clinician-reported outcome measures** in the context of rehabilitating the edentulous maxilla (**category: Peri-implant tissue health**). All outcomes must be assessed, and multiple domains can be selected per outcome.

	Patient selection	Diagnostic tools	Treatment planning	Treatment procedure	Complications during treatment procedure	Maintenance	Complications during maintenance
**Biological complications** - Mucositis, peri-implantitis, lack of keratinized mucosa, implant mobility etc...	☐	☐	☐	☐	☐	☐	☐
**History of patient compliance** - Compliers or erratic patients e.g. fewer than 2 appointments per year	☐	☐	☐	☐	☐	☐	☐
**Peri-implant health (implant level)** - No BOP and no bone loss	☐	☐	☐	☐	☐	☐	☐
**Peri-implant health (patient level)** - No implant in patient's mouth depicting BOP and no bone loss	☐	☐	☐	☐	☐	☐	☐
**Peri-implant mucositis** - BOP with or without suppuration and deep PD; no bone loss	☐	☐	☐	☐	☐	☐	☐
**Peri-implant suppuration** - Determination of suppuration upon gentle probing (site evaluation suppuration yes/no)	☐	☐	☐	☐	☐	☐	☐
**Peri-implantitis** - BOP and/or suppuration, with or without deep PD; with bone loss	☐	☐	☐	☐	☐	☐	☐

* 6. Please indicate in which stage(s) of the GCCG workflow are relevant for the following **subjective clinician-reported outcome measures** (clinicians' perception) in the context of rehabilitating the edentulous maxilla. All outcomes must be assessed, and multiple domains can be selected per outcome.

	Patient selection	Diagnostic tools	Treatment planning	Treatment procedure	Complications during treatment procedure	Maintenance	Complications during maintenance
**Clinician's treatment success** - Treatment success assessed by the clinician via VAS (0-100) or a NRS (0-10)	☐	☐	☐	☐	☐	☐	☐
**Prosthodontic maintenance events/complications** - Perception of the clinician regarding the maintenance of the prosthesis and complications	☐	☐	☐	☐	☐	☐	☐

Please remember to click the '**Done**' button to submit your response!
