# Supplementary material for: 1st Global Consensus for Clinical Guidelines: Identifying a Core Outcome Set for Implant Dentistry in Edentulous Maxilla Rehabilitation
Source: Clin Oral Implants Res. 2026 Feb 24;37(Suppl 30):S108–20. doi: 10.1111/clr.70075 (PMC12930137; doi:10.1111/clr.70075)
Supplement: Supplementary file 4 — Figure S1: Medians and interquartile ranges (IQRs) of patient‐reported outcomes (PROs) scored in the first‐round Delphi survey (92 respondents). Figure S2: Medians and interquartile ranges (IQRs) of objective clinician‐reported outcomes (ClinROs) scored in the first‐round Delphi survey (90 respondents). Figure S3: Medians and interquartile ranges (IQRs) of subjective clinician‐reported outcomes (ClinROs) scored in the first‐round Delphi survey (88 respondents). Figure S4: Medians and interquartile ranges (IQRs) of patient‐reported outcomes (PROs) scored in the second‐round Delphi survey (100 respondents). Figure S5: Medians and interquartile ranges (IQRs) of objective clinician‐reported outcomes (ClinROs) scored in the second‐round Delphi survey (99 respondents): Implant performance; Implant‐supported prosthesis performance; Surgical domain; Peri‐implant tissue health. Figure S6: Medians and interquartile ranges (IQRs) of subjective clinician‐reported outcomes (ClinROs) scored in the second‐round Delphi survey (99 respondents). [file CLR-37-S108-s005.docx]

**Suppl. Figure 1.** Medians and interquartile ranges (IQRs) of patient-reported outcomes (PROs) scored in the first-round Delphi survey (92 respondents)

**Suppl. Figure 2.** Medians and interquartile ranges (IQRs) of objective clinician-reported outcomes (ClinROs) scored in the first-round Delphi survey (90 respondents)

**Suppl. Figure 3.** Medians and interquartile ranges (IQRs) of subjective clinician-reported outcomes (ClinROs) scored in the first-round Delphi survey (88 respondents)

**Suppl. Figure 4.** Medians and interquartile ranges (IQRs) of patient-reported outcomes (PROs) scored in the second-round Delphi survey (100 respondents)

**Suppl. Figure 5.** Medians and interquartile ranges (IQRs) of objective clinician-reported outcomes (ClinROs) scored in the second-round Delphi survey (99 respondents): Implant performance; Implant-supported prosthesis performance; Surgical domain; Peri-implant tissue health.

**Suppl. Figure 6.** Medians and interquartile ranges (IQRs) of subjective clinician-reported outcomes (ClinROs) scored in the second-round Delphi survey (99 respondents)
